# Effects of immunological processes and mild ambient atmosphere alterations on the brain in health and disease

**DOI:** 10.1002/nep3.57

**Published:** 2024-09-11

**Authors:** Piotr Walczak, Xunming Ji, Shen Li, Johannes Boltze

**Affiliations:** 1 Department of Diagnostic Radiology and Nuclear Medicine, Center for Advanced Imaging Research, University of Maryland, Baltimore, Maryland, USA; 2 Beijing Institute of Brain Disorders, Capital Medical University, Beijing, China; 3 School of Life Sciences, University of Warwick, Coventry, UK

Immunological processes in the brain and periphery are well known to affect brain function in health and disease. However, our knowledge about the crosstalk between the immune system and the central nervous system is far from being complete. Ongoing research in this field is uncovering the complex interactions between these “supersystems,” often involving additional organs such as the gut with its microbiome, as well as pathophysiological factors such as hyperglycemia, hypertension, or dyslipidemia.^1–3^ Augmenting our knowledge of these complex interactions has the potential to increase diagnostic and prognostic precision and could lead to the discovery of novel therapeutic targets.

Alterations of the ambient atmosphere have been investigated as potential approaches to improve brain function, primarily under pathological conditions. Prominent examples include hyperbaric oxygenation and the application of noble gases in acute ischemic stroke.^4,5^ Although these approaches have not yet been translated into clinical routine applications, the underlying concepts have remained influential, informing alternative approaches that have been explored subsequently. One of the most intriguing concepts is to provide cerebroprotection by preconditioning, which can be achieved by applying hyperoxygenation as well as hypooxygenation.^6,7^ Since these concepts can be applied easily and widely, they may help to augment our therapeutic arsenal against numerous conditions. However, the mechanisms involved, as well as certain safety concerns, require further detailed investigation.

This issue of *Neuroprotection* features original contributions, including clinical investigations, and reviews that offer new insights into the impact of neuroimmunological processes and mild ambient atmosphere alterations on the brain in health and disease. These studies report promising approaches to improve diagnosis, potential therapeutic targets or concepts to improve brain performance under normal conditions. Key findings presented by these articles will be summarized in this editorial, also briefly discussing the potential future impact of the reported findings.

Olajide et al. provide a comprehensive review on microglial senescence in the context of neurodegeneration. Senescent microglia are characterized by prolonged inflammatory responses and reduced dendritic branching, indicating a reduced capacity to polarize into a pro-regenerative, anti‐inflammatory state which can support functional recuperation.^8^ The review offers detailed insights into alterations of microglial reactivity in neurodegenerative conditions, including changes occurring in senescent microglia such as iron overload and telomere shortening. Interestingly, senescent microglia encompass various states of activation. While they are predominantly proinflammatory the existence of these senescent cells challenges the classical M1/M2 discrimination between pro‐ and anti‐inflammatory microglia. Based on a description of potential senescent microglia markers, the review then summarizes what is known about their role in different neurodegenerative diseases. This is important since identifying senescent microglia is an essential step in dicovering potential new therapeutic targets, and eventually improving the prospects of patients suffering from incurable conditions such as Alzheimer's disease.^9^

Results from a clinical study are reported by Tang et al. who retrospectively investigated the impact of the neutrophil‐to‐lymphocyte (NLR) ratio and hyperglycemia on clinical outcome of stroke patients undergoing recanalization by endovascular thrombectomy. Hyperglycemia at admission was negatively associated with a good functional outcome as defined by functional independence in patients with a low NLR ratio, but not in those exhibiting a high NLR ratio. However, the interaction between NLR ratio and hyperglycemia did not predict hemorrhagic transformation or mortality 3 months after stroke. This suggests that patients with a low NLR might benefit from aggressive antihyperglycemic treatment in acute stroke, although further investigations will be required to confirm this conclusion. Nevertheless, studies such as the one by Tang et al. are important to further augment the impact of recanalization procedures in and after acute ischemic stroke with the aim to improve functional outcome.^10^

The work by Shentu and coworkers identifies a mechanism that induces cognitive impairment in the context of chronic kidney disease (CKD). Initial clinical observations revealed cognitive decline in patients with CKD, as along with increased levels of inflammatory cytokines, in particular interleukin (IL) 6. To further investigate this, mouse hippocampal neuronal (HT22) cells were exposed to high IL‐6 concentrations, causing increased Toll‐like receptor (TLR) 4 expression leading to mitochondria‐dependent apoptosis. Finally, CKD was modeled by bilateral partial nephrectomy in mice. Increased TLR4 and inflammatory cytokine expression was observed in the hippocampi of CKD mice, which also showed cognitive impairment. Finally, TLR‐4 knockdown mitigated hippocampal inflammation and partially increased spatial and nonspatial memory function in these animals. The work not only provides novel mechanistic insights into inflammatory‐driven neurodegeneration with cognitive decline but also informs future translational and clinical research.

Hippocampal injury and cognitive decline were also investigated by Zhao and colleagues, although taking a completely different approach. Female rats were subjected to sleep deprivation followed by sleep recovery with or without combined inhalation of 5% CO_2_. Rats with undisturbed sleep served as controls. Inhalation of 5% CO_2_ fostered recovery of hippocampal glutamate concentrations, enhanced astrocyte‐specific membrane protein glutamate transporter‐1 expression levels and promoted aquaporin‐4 distribution. Moreover, hippocampal neuronal damage was attenuated, as evidenced on a cellular as well as a macroscopic level, the latter by using apparent diffusion coefficient magnetic resonance imaging, eventually leading to improved cognitive function. These interesting findings show that inhalation of 5% CO_2_ may alleviate hippocampal injury and increase cognitive function caused by sleep deprivation, and potentially other conditions.

Mennen and colleagues report on the tolerability of motor‐cognitive training under inspiratory hypoxia in 20 human participants. These probands were either healthy (*n* = 13) or suffered from neuropsychiatric conditions (depression and/or autism spectrum disorders). The work by Mennen et al. build on a recently published concept suggesting that mild to moderate hypoxia could exert beneficial effects on the brain mediated by erythropoietin. ^11^ Indeed, serum erythropoietin levels were elevated in the probands undergoing training. Moderate intensity (3.5 h per day) motor‐cognitive training under inspiratory hypoxia (12% O_2_) over 3 weeks was well tolerated. The study also revealed preliminary evidence for improvements in cognitive performance and physical fitness as well as general wellbeing, although these findings require confirmation in subsequent studies.

Taken together, the current issue of *Neuroprotection* presents intriguing new preclinical and clinical research on neuroinflammatory and inflammatory processes, as well as the effects of ambient atmospheric alterations on brain function in health and disease. It also reveals new mechanistic insights that could inform future translational research aimed at improving diagnostic, prognostic and therapeutic approaches in neurodegenerative conditions.

## Data Availability

Data sharing is not applicable to this article as no new data were created or analyzed in this study.
